# 1011. Geospatial Analysis of Antibiotic Susceptibility in Wisconsin

**DOI:** 10.1093/ofid/ofab466.1205

**Published:** 2021-12-04

**Authors:** Laurel Legenza, Kyle McNair, James P Lacy, Song Gao, Warren Rose

**Affiliations:** University of Wisconsin-Madison, Madison, Wisconsin

## Abstract

**Background:**

The global threat of antimicrobial resistance (AMR) varies regionally. Regional differences may be related to socio-economic factors such as the Area Deprivation Index (ADI) score. Our hypothesis is that AMR spatial distribution is not random.

**Methods:**

Patient level antibiotic susceptibility data was collected from three regionally distinct Wisconsin health systems (UW Health, Fort HealthCare, Marshfield Clinic Health System [MCHS]). Patient addresses were geocoded to coordinates and joined with US Census Block Groups. For each culture source, we included the initial *E. coli* isolate per patient per year with a patient address in Wisconsin. Percent susceptibility was calculated by block group. Spatial autocorrelation was determined by Global Moran’s I, which quantifies the attribute being analyzed as spatially dispersed, randomly distributed, or clustered by a range of −1 to +1. Linear regression correlated ADI to susceptibility. Hot spot analysis identified blocks with statistically significant higher and lower susceptibility (Figure 1).

Figure 1. Geographic example of hot spot analysis and interpretation.

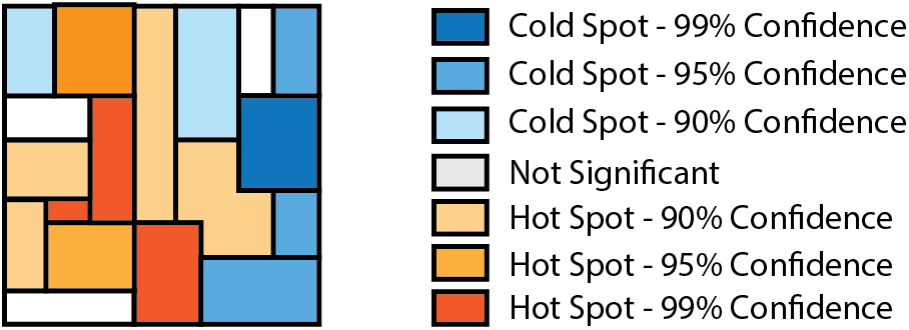

**Results:**

The UW Health results included more urban areas, more block groups and greater isolate geographic density (n = 44,629 *E. coli*, 2009-2018), compared to Fort HealthCare (n = 6,065 isolates, 2012-2018) and MCHS (50,405 isolates, 2009-2018). A positive spatially clustered pattern was identified from the UW Health data for ciprofloxacin (Moran’s I = 0.096, p = 0.005) and trimethoprim/sulfamethoxazole (TMP/SMX) susceptibility (Moran’s I = 0.180, p < 0.001; Figures 2-3). Fort HealthCare and MCHS distribution was likely random for TMP/SMX and ciprofloxacin by Moran’s I. Linear regression of ADI (scale 1-10, least to most disadvantaged) and susceptibility did not find significance, but susceptibility was lower in more disadvantaged block groups. At the local level, we identified hot and cold spots with 90%, 95%, and 99% confidence, with more hot spots in rural regions.

Figure 2. Results from Moran’s Index analysis identifying geographically clustered ciprofloxacin susceptibility results.

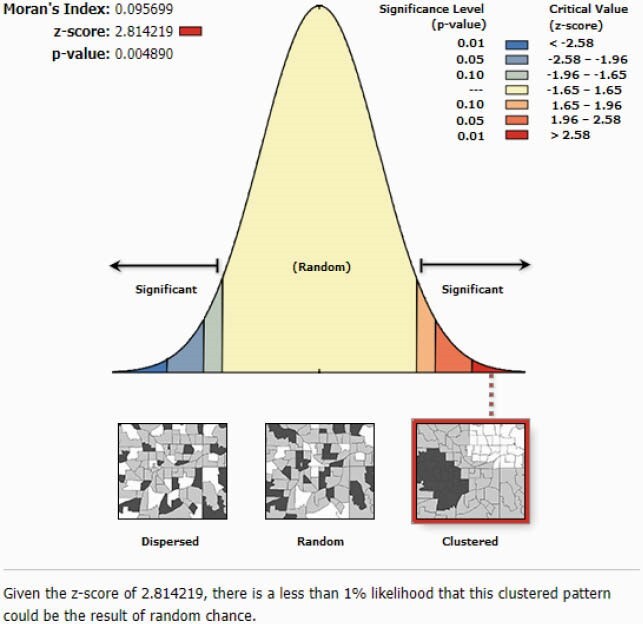

Figure 3. Results from Moran’s Index analysis identifying geographically clustered sulfamethoxazole/trimethoprim susceptibility results.

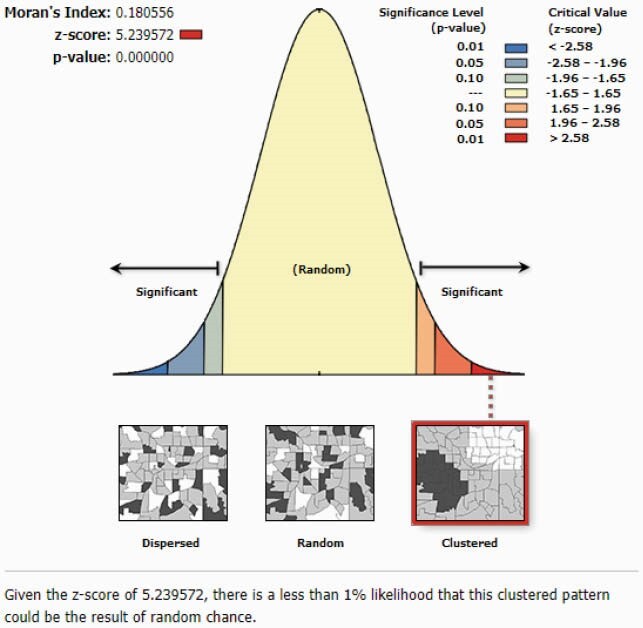

**Conclusion:**

Overall, Moran’s I analysis is more able to identify a clustered pattern in urban versus rural areas. Yet, the local hot spot results indicate that variations in antibiotic susceptibility may be more common in rural areas. The results are limited to data from patients with access to the health systems included.

**Disclosures:**

**Warren Rose, PharmD, MPH**, **Merck** (Grant/Research Support)**Paratek** (Grant/Research Support, Advisor or Review Panel member)

